# Livestock-associated Methicillin-Susceptible *Staphylococcus aureus* ST398 Infection in Woman, Colombia

**DOI:** 10.3201/eid1710.110638

**Published:** 2011-10

**Authors:** J. Natalia Jiménez, Lázaro A. Vélez, José R. Mediavilla, Ana M. Ocampo, Johanna M. Vanegas, Erika A. Rodríguez, Barry N. Kreiswirth, Margarita M. Correa

**Affiliations:** Universidad de Antioquia, Medellín, Colombia (J.N. Jiménez, L.A. Vélez, A.M. Ocampo, J.M. Vanegas, E.A. Rodríguez, M.M. Correa);; University of Medicine and Dentistry of New Jersey, Newark, New Jersey, USA (J.R. Mediavilla, B.N. Kreiswirth)

**Keywords:** MSSA, livestock-associated *S. aureus*, ST398, Colombia, human, letter

**To the Editor:** *Staphylococcus aureus* causes health care– and community-associated infections worldwide in humans and animals. It also asymptomatically colonizes a large proportion (20%–60%) of otherwise healthy individuals. In recent years, various countries have reported an increasing number of humans infected with livestock-associated *S. aureus* multilocus sequence type (ST) 398, which suggests that this strain is emerging in community and health care settings (*1*). Methicillin-resistant *S. aureus* (MRSA) ST398 has received particular attention as a causative agent of infection in pigs, dogs, horses, cattle, and poultry. Colonization and infection in humans have also been described in Europe (*2*), Asia (*3*), Canada (*4*), and the United States (*5*), particularly among persons with frequent exposure to animals, such as farmers, veterinarians, and their household members. However, infections with MRSA ST398 and methicillin-susceptible *S. aureus* (MSSA) ST398 have recently been described in persons with no history of contact with livestock (*6**–**10*).

We report infection of a woman with MSSA ST398 in Colombia, South America. On November 3, 2009, this 82-year-old woman was admitted to the emergency unit of the Hospital Universitario San Vicente Fundación in Medellín, reporting a 15-day history of fever, dyspnea, and pain in her left leg. She lived in a rural area and reported previous contact with dogs and chickens. Her medical history included diabetes mellitus, hypertension, valvular heart disease, and chronic arterial occlusive disease. Four months earlier she had received a femoro–popliteal vascular prosthetic graft in her left leg.

At the time of admission, blood culture was requested, and intravenous vancomycin (1 g every 12 hours) and piperacillin/tazobactam (4.5 g every 8 hours) were empirically administered. *S. aureus* was subsequently isolated from blood culture, and antimicrobial drug susceptibility was assessed in accordance with Clinical Laboratory Standards Institute guidelines by using a Vitek 2 instrument (bioMérieux, Marcy l’Etoile, France). The isolate was susceptible to methicillin, rifampin, and vancomycin but resistant to clindamycin, erythromycin, gentamicin, levofloxacin, minocycline, moxifloxacin, tetracycline, and trimethoprim/sulfamethoxazole. Additional laboratory results showed an elevated leukocyte count with predominant polynuclear neutrophils and increased C-reactive protein levels (21.2 mg/L).

Angiography of the left femoro–popliteal segment showed a collection surrounding the entire vascular prosthetic graft, which was presumed to be the bacteremic focus. Accordingly, rifampin (600 mg every 12 hours) was added to the regimen, the femoro-popliteal graft was surgically removed, the collection was drained, and the limb was amputated. After the surgery, cephradine was administered for 14 days, after which clinical signs and symptoms of bacteremia resolved completely, and the patient was discharged from the hospital.

The blood culture isolate was subsequently confirmed as *S. aureus* by PCR with primers directed to the *nuc* gene. Genes encoding the following virulence factors were also evaluated by PCR, but none were detected: Panton-Valentine leukocidin, arginine catabolic mobile element, staphylococcal enterotoxins A–E, exfoliating toxins A and B, and toxic shock syndrome toxin 1. Genotypic analysis indicated that the isolate belonged to multilocus ST398 (allelic profile 3-35-19-2-20-26-39) and *spa* type t571 (eGenomics *spa* type 109); pulsed*-*field gel electrophoresis with *Sma*I digestion yielded no results, as described previously for ST398 (*1*).

This report documents the emergence of human infection caused by MSSA *spa* type t571 ST398 in South America. Despite being about only 1 case, this report nevertheless highlights the changing epidemiology of *S. aureus* within the region. The study was limited by the inability to sample animals from a surrounding farm to determine the potential for zoonotic spread of *S. aureus* in domestic environments. Notably, *spa* type t571 ST398 has been found recently in MSSA carriage isolates from New York City (*6*), the Dominican Republic (*6*), and the Amazonian region of French Guiana (*9*) and in clinical MSSA isolates from the Netherlands (*7*), People’s Republic of China (*8*), and France (*10*). Given the patients’ absence of contact with livestock in most of these reports, transmission of MSSA ST398 *spa* type t571 may not be limited to animal exposure, suggesting the possibility of person-to-person spread. Accordingly, our finding reinforces the need to heighten awareness of the transmission and virulence potential of MSSA ST398, particularly in developing countries where understanding of *S. aureus* colonization and transmission dynamics is probably limited. Such information has implications for the design of appropriate control measures to reduce human and animal infections from this emerging pathogen.
